# Identification of the major diacylglycerol acyltransferase mRNA in mouse adipocytes and macrophages

**DOI:** 10.1186/s12858-018-0103-y

**Published:** 2018-12-14

**Authors:** Heping Cao

**Affiliations:** 0000 0004 0404 0958 grid.463419.d U.S. Department of Agriculture, Agricultural Research Service, Southern Regional Research Center, New Orleans, LA 70124 USA

**Keywords:** Adipocytes, Diacylglycerol acyltransferases, Gene expression, Macrophages, Tristetraprolin/Zinc finger protein 36, Tung tree

## Abstract

**Background:**

Triacylglycerols (TAGs) are the major form of energy storage in eukaryotes. Diacylglycerol acyltransferases (DGATs) catalyze the final and rate-limiting step of TAG biosynthesis. Mammalian DGATs are classified into DGAT1 and DGAT2 subfamilies. It was unclear which DGAT was the major isoform expressed in animal cells. The objective was to identify the major DGAT mRNA expressed in cultured mouse adipocytes and macrophages and compared it to that expressed in tung tree seeds.

**Methods:**

qPCR evaluated DGAT mRNA levels in mouse 3 T3-L1 adipocytes and RAW264.7 macrophages and tung tree seeds.

**Results:**

TaqMan qPCR showed that DGAT2 mRNA levels were 10–30 fold higher than DGAT1 in adipocytes and macrophages, and DGAT mRNA levels in adipocytes were 50–100-fold higher than those in macrophages. In contrast, the anti-inflammatory tristetraprolin/zinc finger protein 36 (TTP/ZFP36) mRNA levels were 2–4-fold higher in macrophages than those in adipocytes and similar to DGAT1 in adipocytes but 100-fold higher than DGAT1 in macrophages. SYBR Green qPCR analyses confirmed TaqMan qPCR results. DGAT2 mRNA as the major DGAT mRNA in the mouse cells was similar to that in tung tree seeds where DGAT2 mRNA levels were 10–20-fold higher than DGAT1 or DGAT3.

**Conclusion:**

The results demonstrated that DGAT2 mRNA was the major form of DGAT mRNA expressed in mouse adipocytes and macrophages and tung tree seeds.

## Background

Triacylglycerols (TAGs) are one of the major forms of energy storage in eukaryotes. They also serve as a source of fatty acids for membrane biogenesis and lead to obesity if overly accumulated in adipose tissues [1]. Diacylglycerol acyltransferases (DGATs) catalyze *sn*-1,2-diacylglycerol and a long-chain fatty acyl-CoA into TAG, the rate-limiting step of TAG biosynthesis [2]. DGAT deficiency results in less TAG accumulation [3–5]. DGAT knockout mice exhibit resistance to diet-induced obesity [4, 6], lack milk production [4] and link to a congenital diarrheal disorder [7]. More DGAT enzymatic activity increases TAG content in plants [8–14], animals [15–18] and yeast [19, 20]. Genetic studies have demonstrated that DGAT isoforms are unique in TAG biosynthesis in mice [5] and tung tree [21]. Understanding the specific functions of DGAT isoforms will help engineering plants and microbes with value-added properties and provide guidance for potential intervention and treatment of obesity and related diseases.

Although DGAT1 and DGAT2 perform similar biochemical reaction, they are encoded by distinctive gene families. Generally, DGATs are divided into DGAT1 and DGAT2 subfamilies [21, 22]. DGATs are integral membrane proteins [21, 23] with more than 40% of the total amino acid residues being hydrophobic [24]. We have classified over 100 DGAT protein sequences from 70 organisms into DGAT1 and DGAT2 subfamilies [24]. There are 41 and 16 completely conserved amino acid residues mostly located at the carboxyl termini of DGAT1s and DGAT2s, respectively [24]. DGATs have similar chemical properties and amino acid composition except that DGAT1s are ~ 20 kDa larger than DGAT2s [24]. Recent studies have shown that DGAT3s are present in tung tree (*Vernicia fordii)* [25], yeast (*Rhodotorula glutinis*) [26], *Arabidopsis thaliana* [22], Burning Bush (*Euonymus alatus*) [10], peanut (*Arachis hypogaea*) [27], a bifunctional DGAT in *Acinetobacter calcoaceticus* ADP1 [28], castor bean (*Ricinus communis*) and various other plant species. DGAT3s are different from DGAT1s and DGAT2s [25] because none of the completely conserved residues in DGAT1s (41 residues) and DGAT2s (16 residues) is aligned with DGAT3s [24, 25]. In addition, oil palm (*Elaeis guineensis*) genome has four closely related but differentially expressed DGATs (DGAT1, DGAT2, DGAT3 and WS/DGAT) and at least two members of each of the four DGAT groups are found by in silico analysis [29].

**Fig. 1:**
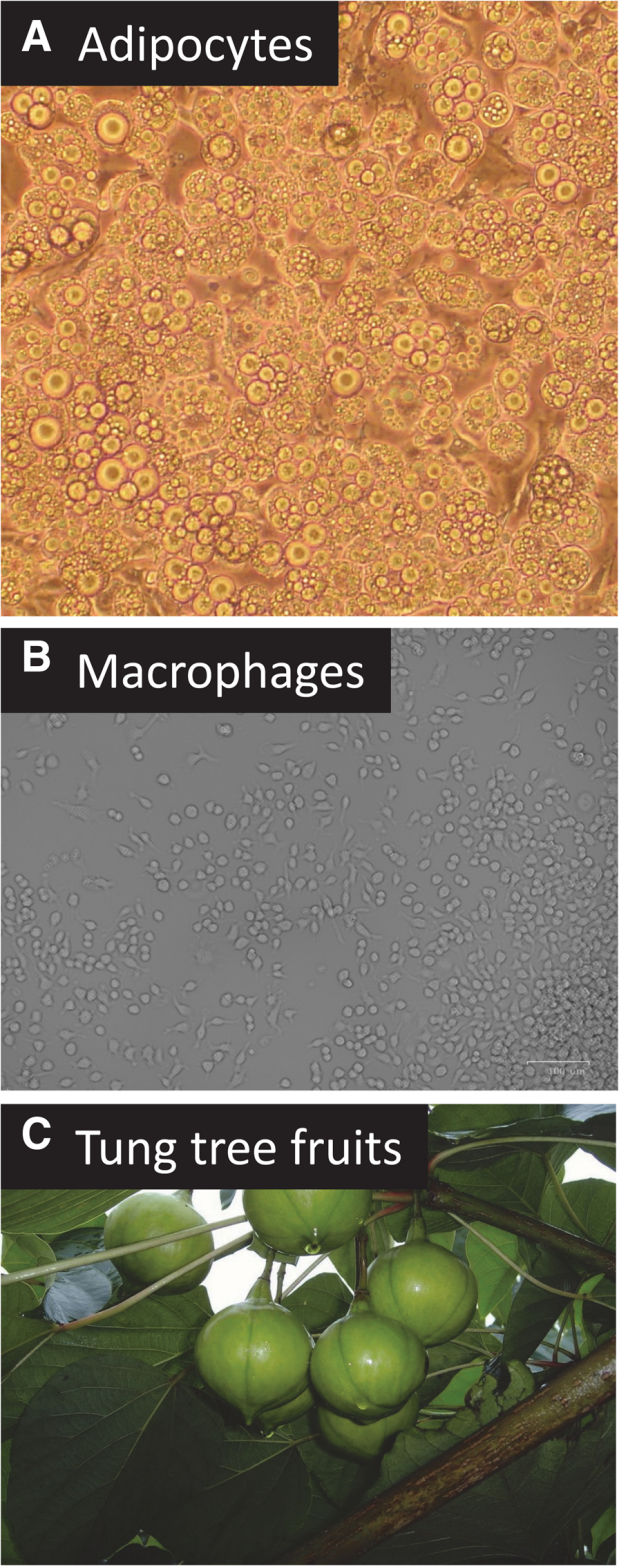
Mouse cells and plant seeds used in the study. **a** Differentiated mouse adipocytes (magnification: 40x). Mouse 3 T3-L1 fibroblasts were differentiated in the induction medium containing insulin, dexamethasone and 1-isobutyl-3-methylxanthine. Microscopic observation indicated that approximately 90% of the cells accumulated lipid drops, an indication of differentiation from preadipocytes to adipocytes. **b** RAW264.7 macrophages (no magnification). **c** Tung tree seeds

Currently, it is not clear which form of DGAT is the major isoform expressed in many mammalian cells. DGAT1 was shown to be highly expressed in mice intestine and plays a crucial role in TAG biosynthesis [30, 31]. In contrast, DGAT2 has been shown to be the major form of DGAT in tung tree seeds [21, 25]. The objective of this study was to identify the major form of DGAT mRNA expressed in mammalian cells using cultured mouse 3 T3-L1 adipocytes and RAW264.7 macrophages and to compare it to DGATs expressed in tung tree seeds to gain insights into which is the major DGAT mRNA in three different types of cells from animal and plant origins (Fig. 1). TaqMan and SYBR Green quantitative real-time PCR (qPCR) assays evaluated mouse DGAT mRNA levels using ribosomal protein L32 (RPL32) gene as an internal reference [32–34]. The positive control of gene expression was using the anti-inflammatory tristetraprolin (TTP)/zinc finger protein 26 (ZFP36) expressed in both mouse 3 T3-L1 adipocytes and RAW264.7 macrophages [35–37]. DGATs of tung tree seeds were also analyzed for comparison. The results reported here conclusively demonstrated that DGAT2 mRNA was the major form of DGAT mRNA expressed in both plant and animal cells tested in this study.

## Methods

### Mouse cell lines and plant seeds

Mouse 3 T3-L1 preadipocytes and RAW264.7 macrophages were purchased from American Type Culture Collection (Manassas, VA). The cells were kept under liquid nitrogen vapor in a Cryogenic Storage Vessel (Thermo Fisher Scientific, Waltham, MA). Tung trees (*Aleurites fordii* Hemsl.) were grown in the American Tung Oil Corporation orchard (Lumberton, Mississippi). Company officer John Corley granted permission of the tung tree fruits for our project. Tung fruits were collected weekly for a total of 11 weeks (seed stage 1–11) beginning 9 weeks after full bloom and 1 month before the initiation of storage oil synthesis as stage 1 seed [25]. Kernels from tung tree seeds from stages 5 to 11 were immediately frozen in liquid N_2_ and stored at − 80 °C before use.

### Chemicals, reagents and equipment

Chemicals, reagents and equipment used in the current study were mostly described previously [38, 39]. Tissue culture reagents were from Gibco BRL (Thermo Fisher). Tissue culture incubator was water jacket CO_2_ incubator, Forma Series II, Model 3100 Series (Thermo Fisher). Tissue culture workstation was Logic+ A2 hood (Labconco, Kansas City, MO). Tissue culture plastic ware was from CytoOne (USA Scientific, Ocala, FL). Cell counting reagent (trypsin blue dye), slides (dual chamber), counter (TC20 Automatic Cell Counter) and microscope (Zoe Florescent Cell Imager) were from Bio-Rad (Hercules, CA). RNA purification reagent (TRIzol), dryer (Integrated SpeedVac System) and quantitation (NanoPhotometer) were from Thermo Fisher, Thermo Fisher and Implen (Munchen, Germany), respectively. cDNA synthesis equipment (DNA Engine, Gradient Cycler, PTC-200) and 8-strip thin-well tubes were from MJ Research (Waltham, MA). cDNA synthesis plate (96-well plate) was from ABgene (Thermo Fisher). cDNA synthesis reagents (SuperScript II reverse transcriptase, oligo (dT)_12–18_ primer, random primers, dNTPs, DTT, RNaseOUT) were from Life Technologies. qPCR equipment (CFX96 real-time system-C1000 Thermal Cycler), reagent (1× iQ SYBR Green Supermix) and accessories (plates and sealing film) were from Bio-Rad. Other chemicals (LPS, gossypol, DMSO, chloroform, hexane, and ethanol) were from Sigma.

### Cell culture

Mouse 3 T3-L1 fibroblasts were maintained in a humidified incubator at 37 °C with 5% CO_2_ in Dulbecco’s modified Eagle’s medium (DMEM) containing 4500 mg/l (25 mM) glucose supplemented with 10% (*v*/v) fetal bovine serum, 100 U/ml penicillin, 100 μg/ml streptomycin, and 2 mM L-glutamine (DMEM+). Adipocyte differentiation was induced as described [40]. Briefly, mouse 3 T3-L1 fibroblasts (about 0.2 million cells/2-ml medium/well) were grown in 6-well plates under the same conditions for 48–60 h and the medium was replaced with fresh DMEM+. After incubation for another 48–60 h, the medium was replaced with differentiation medium containing DMEM+, 1 μg/ml of the recombinant human insulin, 0.25 μM dexamethasone, and 250 μM IBMX. Following incubation for 48–60 h, the differentiation medium was replaced with DMEM+ containing only 1 μg/ml of insulin. After incubation for additional 48–60 h, the medium was replaced with DMEM+ and the cells were grown for an additional 4–6 days. Microscopic observation indicated that approximately 90% of the cells accumulated lipid drops (indication of differentiation from preadipocytes to adipocytes) (Fig. 1a). The adipocytes were then serum-starved for 4 h in DMEM without any supplementation or with % DMSO and collected at various time points for RNA extraction.

Mouse RAW264.7 macrophages were maintained in polystyrene tissue culture flasks as described [41] at 37 °C in a water jacket CO_2_ incubator with 5% CO_2_ in DMEM containing 4.5 mg/ml (25 mM) glucose supplemented with 10% (v:v) fetal bovine serum, 100 units/ml penicillin, 100 μg/ml streptomycin, and 2 mM L-glutamine. RAW macrophages were dissociated from the flask with a cell scraper, stained with equal volume of 0.4% trypsin blue dye before counting the number of live cells with a TC20 Automatic Cell Counter. RAW cells (0.5 ml) were subcultured at approximately 1 × 10^5^ cells/ml density in 12-well tissue culture plates. The macrophages were subcultured for two days before being added with 0.1–1% DMSO and collected at various time points for RNA extraction. Mouse RAW macrophages were observed routinely under a Zoe Florescent Cell Imager before and under treatment (Fig. 1b).

### RNA extraction

Mouse cells in culture plates were washed with 1 ml 0.9% NaCl twice and lysed directly with 1 ml of TRIzol reagent. RNA was isolated according to the manufacturer’s instructions. Tung seeds (Fig. 1c) were ground into powder under liquid nitrogen. Total RNAs were isolated by Spectrum Plant Total RNA Kit as described [32]. RNA concentrations and quantity were determined using RNA 6000 Nano Assay Kit and the Bioanalyzer 2100 according to the manufacturer’s instructions with RNA 6000 Ladder as the standards [35]. RNA concentrations were also quantified with an Implen NanoPhotometer. The RNA preparations were of high quality as determined by high rRNA ratio (28S/18S = 1.9) and the RNA integrity number (RIN = 8.7).

### Quantitative real-time PCR analysis

TaqMan and SYBR Green qPCR assays generally followed the MIQE guidelines [42]. Unique PCR primers and TaqMan probes for each DGAT isoform were designed using Primer Express software. They were synthesized by Biosearch Technologies, Inc. The forward primers, TaqMan probes (TET – BHQ1) and reverse primers, respectively, are described in Table 1.

The cDNAs were synthesized from total RNAs using SuperScript II reverse transcriptase. The cDNA synthesis mixture (20 μl) contained 2.5–5 μg total RNA, 2.4 μg oligo (dT)_12–18_ primer, 0.1 μg random primers, 500 μM dNTPs, 10 mM DTT, 40 units RNaseOUT and 200 units SuperScript II reverse transcriptase in 1X first-strand synthesis buffer. The cDNA synthesis reactions were at 42 °C for 50 min. The cDNA samples were stored in − 80 °C freezer and diluted with water to 1 ng/μl before analyses.

TaqMan qPCR mixtures contained 5 ng of cDNAs except Table 2 using 25 ng cDNAs, 200 nM each of the forward primer, reverse primer and TaqMan probe, and Absolute QPCR Mix [35]. The thermal cycling conditions for TaqMan qPCR were 2 min at 50 °C and 15 min at 95 °C, followed by 50 cycles at 95 °C for 15 s and 60 °C for 60 s. SYBR Green qPCR mixtures contained 5 ng of cDNAs, 200 nM each of forward primer and reverse primer and iQ SYBR Green Supermix. The thermal cycle conditions for SYBR Green qPCR were 3 min at 95 °C, followed by 40 cycles at 95 °C for 10 s, 65 °C for 30 s and 72 °C for 30 s. The qPCR reactions were performed in 96-well clear plates sealed by adhesives with an ABI Prism 7700 real time PCR instrument or a CFX96 real-time system-C1000 Thermal Cycler. Both types of equipment were shown to be reliable for qPCR analyses [34].

### Data analysis

The fold change in expression was determined by the ΔΔC_T_ method of relative quantification [43]. First, the mean and standard deviation of the cycle of threshold (C_T_) was obtained from independent samples. Second, the first delta C_T_ value (ΔC_T_) was obtained by subtracting the C_T_ values of the internal reference control, mouse 60S ribosome protein 32 (Rpl32) or tung tree 60s Rpl19b from the mean C_T_ values of the target mRNAs (ΔC_T_ = C_TTarget_ - C_Tref_). The selection of these two internal reference controls was based on our previous studies showing that RPL32 and RPL19b were the most stably expressed genes in the mouse cells and tung tree tissues, respectively [33, 44]. Third, the second delta C_T_ value (ΔΔC_T_) was obtained by subtracting the ΔC_T_ of the calibrator, time-point 0 min, TTP control, adipocytes, or DGAT1 in tune tree seed from the ΔC_T_ of the target mRNAs (ΔΔC_T_ = ΔC_TTarget_ - ΔC_Tcal_). Fourth, the fold change in expression was obtained using the eq. (2^-ΔΔCT^). Finally, the ratio of Dgat2’s fold change/Dgat1’s fold change over 2, i.e., [(2^-ΔΔCT^) of Dgat2/[(2^-ΔΔCT^) of Dgat1 > 2], was used as the threshold for significant differences in gene expression [45–48].

## Results

### Cycle of threshold of RPL32, TTP and DGAT mRNAs in mouse adipocytes

Mouse 3 T3-L1 fibroblasts were differentiated into adipocytes by insulin, dexamethasone, and 1-isobutyl-3-methylxanthine (IBMX). Microscopic observation showed that almost all adipocytes accumulated lipid droplets (indication of differentiation from preadipocytes to adipocytes) (Fig. 1a). The expression level of a gene coding for adipocyte-specific cytokine, adiponectin (ADIPOQ) was extremely abundant and close to half of the house-keeping RPL32 mRNA levels in the adipocytes (Table 2). Adiponectin mRNA levels were 49, 37, 55 and 53% of RPL32 mRNA levels in adipocytes collected at 0, 30, 60 and 90-min, respectively (Table 2), in agreement with previous results showing that ADIPOQ mRNA is one of the most abundant adipokines [49]. ADIPOQ expression data support the microscopic observation that the adipocytes were properly differentiated under the experimental conditions.

### Time-course of RPL32, TTP and DGAT mRNAs in mouse adipocytes

**Fig. 2:**
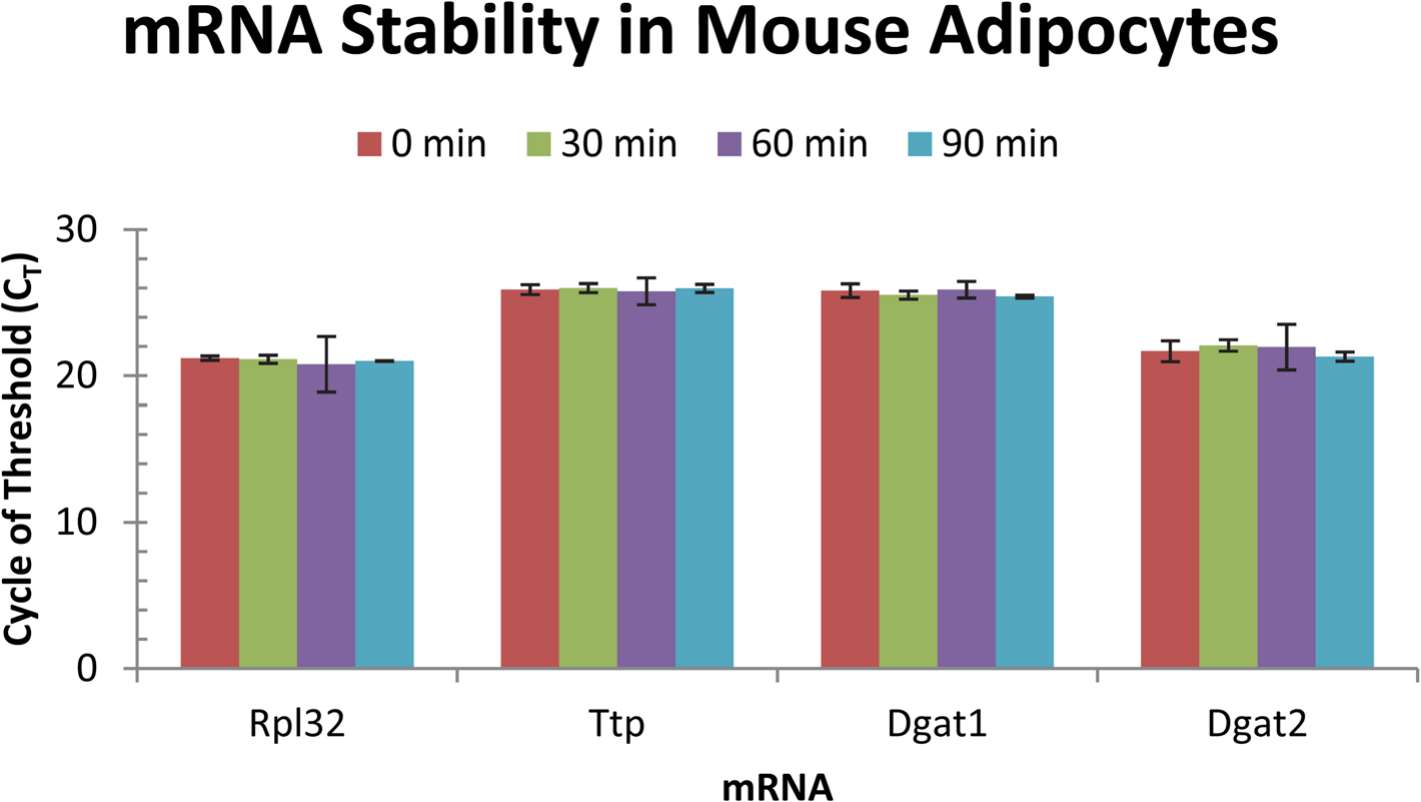
TTP and DGAT mRNAs were stable in mouse adipocytes. The differentiated 3 T3-L1 adipocytes were serum-starved for 4 h in DMEM without any supplementation and collected at various time points (0, 30, 60, and 90 min after 4-h starvation) for RNA extraction and cDNA synthesis. TaqMan qPCR assay evaluated Rpl32, Ttp, Dgat1, and Dgat2 gene expression. The data represent the mean and standard deviation of four assays

The adipocytes were serum-starved in DMEM without any supplementation for 4 h before collecting cells at 0, 30, 60, and 90 min. Total RNAs were extracted from the cells, converted into cDNAs, and analyzed by TaqMan qPCR method using primers and probes as shown in Table 1. The C_T_ values for each of the mRNA tested including the reference gene RPL32, positive control gene TTP, and DGAT1 and DGAT2 genes were very similar among the cells collected at various time-points following starvation (Fig. 2). These qPCR data were used to calculate mRNA levels in mouse cells using the formula (2^-ΔΔ**CT**^). RPL32 mRNA levels were similar among the four time points with only slightly increased in the 90 min sample after 4-h serum-starvation. The differences of gene expression among the cells collected at various time-points after serum starvation were less than 2-fold difference, i.e., the ratio (2^-ΔΔ**CT**^) is < 2. Therefore, these genes were expressed relatively stable in mouse adipocytes under the experimental conditions.

**Table 1:** Nucleotide sequence information of qPCR primers and TaqMan probes

Species	mRNA	Name	Accession no.	Amplicon (bp)	Forward primer (5′ to 3′)	TaqMan probe (5′ to 3′)	Reverse primer (5′ to 3′)
Mouse	Dgat1	Diacylglycerol acyltransferase 1	NM_010046	61	CGTGGGCGACGGCTACT	ATCTGAGGTGCCATCG	TGAGCTGAACAAAGAATCTTGCA
	Dgat2	Diacylglycerol acyltransferase 2	NM_026384	62	CTGGCTGATAGCTGTGCTCTACTT	CTGGCTGGCATTTG	CTTTCTTGGGCGTGTTCCA
	Ttp	Tristetraprolin/Zfp36	NM_011756	70	GGTACCCCAGGCTGGCTTT	AACTCAATATAATCCTGCCTTAGCCTT	ACCTGTAACCCCAGAACTTGGA
	Adipoq	Adiponectin	NM_009605	180	CCCTCCACCCAAGGGAACTT	TGCAGGTTGGATGGCAGGCATC	TCAGCTCCTGTCATTCCAACAT
	Rpl32	60S ribosome protein L32	NM_172086	66	AACCGAAAAGCCATTGTAGAAA	AGCAGCACAGCTGGCCATCAGAGTC	CCTGGCGTTGGGATTGG
Tung tree	Dgat1	Diacylglycerol acyltransferase 1	DQ356680	70	TGGTTGCTCCCACATTGTGT	ACCAGCCAAGTTATCCTCGAACTGCATCC	ACCACCCAACCCTTTCGAA
	Dgat2	Diacylglycerol acyltransferase 2	DQ356682	80	TGCATGTCGTGGTGGGTAGA	AAGCAAAATCCACAGCCTACCGCTGAA	TCTCTGTACTTCCGCAACCT
	Dgat3	Diacylglycerol acyltransferase 3	KC993814	64	AAGTGCAGGGACGGTCCTAA	TCAGGGTCAATGCTG	TGGGTGGAGTTGTGATTGGA
	Rpl19b	60S Ribosomal protein L19b	FJ362591	70	GCGGAGAATGCGTGTTCTG	CCTGCTGCGCAAATACCGGGAA	CATGTGCTTGTCAATTTTTTTGG

**Table 2:** Relative abundance of Rpl32 and Adipoq mRNAs in mouse adipocytes

Time (min)	mRNA	C_T_ ± SD (*n* = 2–4)	ΔC_T_ (C_TAdipoq_ - C_TRpl32_)	Fold (2^-ΔCT^)
0	Rpl32	18.08 ± 0.13	0.00	1.00
	Adipoq	19.10 ± 0.14	1.03	0.49
30	Rpl32	17.91 ± 0.16	0.00	1.00
	Adipoq	19.34 ± 0.28	1.43	0.37
60	Rpl32	19.40 ± 0.10	0.00	1.00
	Adipoq	20.27 ± 0.00	0.87	0.55
90	Rpl32	18.20 ± 0.09	0.00	1.00
	Adipoq	19.12 ± 0.17	0.92	0.53

a

**Table 3:** Relative abundance of TTP and DGAT mRNAs in mouse adipocytes

Time (min)	mRNA	C_T_ ± SD (*n* = 4)	Fold (2^-ΔΔCT^)	Fold (Dgat2/Dgat1)
0	Ttp	25.89 ± 0.34	1.00	
	Dgat1	25.82 ± 0.47	1.05	1.00
	Dgat2	21.68 ± 0.71	18.51	17.63
30	Ttp	26.00 ± 0.31	1.00	
	Dgat1	25.52 ± 0.28	1.39	1.00
	Dgat2	22.07 ± 0.39	15.24	10.96
60	Ttp	25.78 ± 0.92	1.00	
	Dgat1	25.89 ± 0.57	0.93	1.00
	Dgat2	21.96 ± 1.56	14.12	15.18
90	Ttp	25.98 ± 0.28	1.00	
	Dgat1	25.42 ± 0.10	1.47	1.00
	Dgat2	21.31 ± 0.32	25.46	17.32

**Table 4:** Relative abundance of TTP and DGAT mRNAs in DMSO-treated mouse adipocytes

Time (min)	mRNA	C_T_ ± SD (*n* = 4)	Fold (2^-ΔΔCT^)	Fold (Dgat2/Dgat1)
0	Ttp	26.43 ± 0.09	1.00	
	Dgat1	25.53 ± 0.09	1.87	1.00
	Dgat2	21.24 ± 0.11	36.50	19.52
30	Ttp	25.92 ± 0.12	1.00	
	Dgat1	25.71 ± 0.19	1.16	1.00
	Dgat2	21.16 ± 0.36	27.10	23.36
60	Ttp	26.66 ± 0.38	1.00	
	Dgat1	25.60 ± 0.05	2.08	1.00
	Dgat2	20.86 ± 0.32	55.72	26.79
90	Ttp	26.92 ± 0.37	1.00	
	Dgat1	25.80 ± 0.20	2.17	1.00
	Dgat2	21.32 ± 0.30	48.50	22.35
120	Ttp	26.61 ± 0.01	1.00	
	Dgat1	25.89 ± 0.10	1.65	1.00
	Dgat2	22.63 ± 0.10	15.78	9.56

**Table 5:** Relative abundance of TTP and DGAT mRNAs in DMSO-treated mouse macrophages

Time (min)	mRNA	C_T_ ± SD (*n* = 4)	Fold (2^-ΔΔCT^)	Fold (Dgat2/Dgat1)
0	Ttp	24.94 ± 0.25	1.00	
	Dgat1	31.63 ± 0.30	0.01	1.00
	Dgat2	27.60 ± 1.01	0.16	16.00
30	Ttp	24.90 ± 0.17	1.00	
	Dgat1	31.13 ± 0.23	0.01	1.00
	Dgat2	27.27	0.19	19.00
60	Ttp	24.34 ± 0.10	1.00	
	Dgat1	31.79 ± 0.14	0.01	1.00
	Dgat2	27.01 ± 0.60	0.16	16.00
120	Ttp	24.93 ± 0.25	1.00	
	Dgat1	31.89 ± 0.27	0.01	1.00
	Dgat2	27.01 ± 2.06	0.24	24.00
240	Ttp	25.09 ± 0.14	1.00	
	Dgat1	31.84 ± 0.20	0.01	1.00
	Dgat2	28.48 ± 0.72	0.10	10.00

**Table 6:** Relative abundance of TTP and DGAT mRNAs in DMSO-treated mouse macrophages by SYBR Green qPCR

Time (h)	mRNA	C_T_ ± SD (*n* = 4–8)	Fold (2^-ΔΔCT^)	Fold (Dgat2/Dgat1)
2	Ttp	24.40 ± 0.90	1.00	
	Dgat1	30.51 ± 0.68	0.004	1.00
	Dgat2	28.39 ± 0.93	0.02	5.00
8	Ttp	26.88 ± 0.93	1.00	
	Dgat1	31.69 ± 1.97	0.04	1.00
	Dgat2	28.90 ± 1.43	0.25	6.17
24	Ttp	24.76 ± 1.44	1.00	
	Dgat1	31.10 ± 1.53	0.01	1.00
	Dgat2	27.86 ± 0.65	0.12	12.00

**Table 7:** Relative abundance of TTP and DGAT mRNAs between mouse cells

mRNA	Time (min)	Cell	C_T_ ± SD (*n* = 4)	Fold (2^-ΔΔCT^)
Ttp	0	Adipocytes	26.43 ± 0.09	1.00
		Macrophages	24.94 ± 0.25	1.92
	30	Adipocytes	25.92 ± 0.12	1.00
		Macrophages	24.90 ± 0.17	1.48
	60	Adipocytes	26.66 ± 0.38	1.00
		Macrophages	24.34 ± 0.10	3.51
	120	Adipocytes	26.61 ± 0.01	1.00
		Macrophages	24.93 ± 0.25	1.34
Dgat1	0	Adipocytes	25.53 ± 0.09	1.00
		Macrophages	31.63 ± 0.30	0.01
	30	Adipocytes	25.71 ± 0.19	1.00
		Macrophages	31.13 ± 0.23	0.02
	60	Adipocytes	25.60 ± 0.05	1.00
		Macrophages	31.79 ± 0.14	0.01
	120	Adipocytes	25.89 ± 0.10	1.00
		Macrophages	31.89 ± 0.27	0.01
Dgat2	0	Adipocytes	21.24 ± 0.11	1.00
		Macrophages	27.60 ± 1.01	0.01
	30	Adipocytes	21.16 ± 0.36	1.00
		Macrophages	27.27 ± 0.00	0.02
	60	Adipocytes	20.86 ± 0.32	1.00
		Macrophages	27.01 ± 0.60	0.01
	120	Adipocytes	22.63 ± 0.10	1.00
		Macrophages	27.01 ± 2.06	0.02

**Table 8:** Relative abundance of DGAT mRNAs in tung tree seeds

Seed stage	mRNA	C_T_ ± SD (*n* = 3)	Fold (2^-ΔΔCT^)	Fold (Dgat3/Dgat2)
5	Dgat1	26.62 ± 0.47	1.00	
	Dgat2	23.58 ± 0.98	8.21	1.00
	Dgat3	25.10 ± 1.42	2.87	0.35
6	Dgat1	26.28 ± 0.80	1.00	
	Dgat2	23.58 ± 0.98	5.15	1.00
	Dgat3	25.57 ± 0.60	1.64	0.32
7	Dgat1	26.10 ± 0.69	1.00	
	Dgat2	23.63 ± 1.03	5.56	1.00
	Dgat3	26.17 ± 1.29	0.95	0.17
8	Dgat1	26.21 ± 0.46	1.00	
	Dgat2	23.69 ± 1.18	5.75	1.00
	Dgat3	26.19 ± 0.54	1.01	0.18
9	Dgat1	26.94 ± 1.00	1.00	
	Dgat2	22.62 ± 1.83	20.04	1.00
	Dgat3	25.83 ± 0.75	2.16	0.11
10	Dgat1	27.07 ± 1.88	1.00	
	Dgat2	22.95 ± 0.60	17.33	1.00
	Dgat3	25.44 ± 0.52	3.11	0.18
11	Dgat1	26.79 ± 0.36	1.00	
	Dgat2	23.12 ± 0.76	12.74	1.00
	Dgat3	25.60 ± 0.40	2.28	0.18

### Relative abundance of TTP and DGAT mRNAs in mouse adipocytes

Table 3 shows the relative expression abundance of TTP and DGAT genes in mouse adipocytes. At the start of collecting cells (0 min after 4-h serum starvation), DGAT1 mRNA levels were 1-fold of TTP mRNA. Importantly, DGAT2 mRNA levels were 19-fold of TTP and 18-fold of DGAT1 (Table 3). Similarly, DGAT1 mRNA levels were 1-fold of TTP mRNA, but DGAT2 mRNA levels were 15-fold of TTP and 11-fold of DGAT1 in adipocytes collected at 30 min (Table 3). In adipocytes collected at 60 min, DGAT1 mRNA levels were 1-fold of TTP mRNA, but DGAT2 mRNA levels were 14-fold of TTP and 15-fold of DGAT1 (Table 3). At the last time point (90 min), DGAT2 mRNA levels were 26-fold of TTP and still 17-fold of DGAT1 (Table 3). These data demonstrated that DGAT2 mRNA levels were at least 10-fold higher than both DGAT1 and TTP and that DGAT1 and TTP mRNA levels were similar in the adipocytes regardless of culture medium conditions might being changed over time during the course of cell culture.

### Relative abundance of TTP and DGAT mRNAs in DMSO-treated mouse adipocytes

Since dimethylsulfoxide (DMSO) was used frequently as a solvent for many chemicals in cell cultures and was shown to increase lipolysis under high concentration [50], we analyzed the relative levels of TTP and DGAT gene expression by TaqMan qPCR in the same way using adipocytes treated with 0.1% DMSO (Table 4). DGAT2 mRNA levels were 20-, 23-, 27-, 22- and 10-fold of DGAT1, and were 37-, 27-, 56-, 49- and 16-fold of TTP in adipocytes collected at 0, 30, 60, 90, and 120 min, respectively (Table 4). DGAT1 mRNA levels were approximately 2-fold or less of TTP in the DMSO-treated adipocytes collected at various times (Table 4). Addition of 0.1% DMSO in the culture medium increased the ratio of DGAT2 over DGAT1 mRNAs in the mouse adipocytes, mainly due to increased expression of DGAT2 in the cells (Table 4 vs. Table 3). These data confirmed the conclusion from the above experiments without DMSO in the culture medium that DGAT2 mRNA levels were at least 10-fold higher than both DGAT1 and TTP in mouse 3 T3-L1 adipocytes.

### Relative abundance of TTP and DGAT mRNAs in DMSO-treated mouse macrophages

To confirm if DGAT2 mRNA is a major form of DGAT mRNA in different types of cells, the relative expression levels of these same genes were analyzed by TaqMan qPCR in 0.1% DMSO-treated mouse RAW264.7 macrophages, a cell type with minimal lipid biosynthesis. DGAT1 mRNA levels were only 1% of TTP in macrophages collected at each of the five time points (0, 30, 60, 90, 120, 240 min) (Table 5). DGAT2 mRNA levels were 16, 19, 16, 24, and 10% of TTP mRNA levels in macrophages collected at 0, 30, 60, 90, 120, and 240 min, respectively (Table 5). DGAT2 mRNA levels were 16-, 19-, 16-, 24- and 10-fold of DGAT1 mRNA levels in macrophages collected at 0, 30, 60, 90, 120, and 240 min, respectively (Table 5). These data further confirmed the conclusion from the above experiments from adipocytes that DGAT2 mRNA was the major DGAT mRNA in the cultured mouse cells, although DGAT mRNA levels were much lower than TTP in macrophages.

### Relative abundance of TTP and DGAT mRNAs in DMSO-treated mouse macrophages by SYBR green qPCR assay

To prove that DGAT2 mRNA was the major DGAT gene expressed in the mouse cells from TaqMan qPCR assays, SYBR Green qPCR was used to confirm the expression levels of these four genes in DMSO-treated mouse macrophages. As shown in Table 6, DGAT1 mRNA levels were 0.4, 4 and 1% of TTP in RAW264.7 macrophages collected in 2, 8, and 24 h, respectively. Similarly, DGAT2 mRNA levels were 2, 25 and 12% of TTP in RAW264.7 macrophages collected in 2, 8, and 24 h, respectively. Again, DGAT2 mRNA levels were 5-, 6- and 12-fold of DGAT1 (Table 6). These data further confirmed the conclusion from previous experiments using TaqMan qPCR assay that DGAT2 mRNA was the major DGAT mRNA in the mouse cells, although the fold of difference between DGAT2 and DGAT1 mRNAs by SYBR Green qPCR assay was less than that from TaqMan qPCR assay. These results also showed that DGAT2 mRNA was the major form of DGAT transcript in mouse macrophages under higher DMSO concentration for longer treatment time, a similar trend from TaqMan qPCR assays using macrophages cultured under lower DMSO concentration for shorter treatment.

### Relative abundance of TTP and DGAT mRNAs between mouse adipocytes and macrophages

TTP and DGAT mRNA levels were compared between the two types of cells used in the analysis. Table 7 shows that TTP mRNA levels in macrophages were 2-fold of those in adipocytes in 0-min sample. The ratio was similar in 30-min sample (2-fold) but increased to 4-fold in 60-min sample and returned to 1-fold in 120-min sample (Table 7). DGAT1 and DGAT2 mRNA levels in macrophages were only 1–2% of those in adipocytes in 0, 30, 60, and 120 min samples (Table 7).

### Relative abundance of DGAT mRNAs in developing tung seeds

To evaluate whether DGAT2 mRNA was the major form of DGAT expressed in other eukaryotes, relative expression of three DGAT genes in the developing seeds from three separate field-grown tung trees was measured by TaqMan qPCR using well-characterized RPL19b as an internal reference control [33]. In tung tree seeds with active oil accumulation (stage 5–11 seeds), DGAT2 mRNA levels were approximately 8-, 5-, 6-, 6-, 20-, 17- and 13-fold of DGAT1 in the seeds collected at 5, 6, 7, 8, 9, 10, and 11 stages, respectively (Table 8). DGAT3 mRNA levels were 0.4-, 0.3-, 0.2-, 0.2-, 0.1-, 0.2- and 0.2-fold of DGAT2 in the seeds collected at 5, 6, 7, 8, 9, 10, and 11 stages, respectively (Table 8). These data support the concept that DGAT2 mRNA is the major form of DGAT expressed in both oil-filling tung tree seeds and the two cultured mouse cells.

## Discussion

Diacylglycerol acyltransferases (DGATs) catalyze the final and rate-limiting step of TAG biosynthesis. Animals contain two isoforms but plants and yeast contain three isoforms of DGATs [21, 26, 27, 51, 52]. Genetic studies have demonstrated that DGAT isoforms have non-redundant functions in TAG biosynthesis in mice [5] and tung tree [21, 53]. DGAT2 has been shown to be the major form of DGAT in tung tree seeds [21, 25]. However, it was not clear which form of DGAT was the major isoform expressed in cultured mammalian cells.

The major finding of this report is that DGAT2 mRNA is the major form of DGAT expressed in mouse adipocytes and macrophages, similar to the oil-rich seeds of tung tree. Several lines of evidence support this conclusion. First, DGAT2 mRNA levels were much higher than DGAT1 mRNA levels in mouse 3 T3-L1 adipocytes. Second, DGAT2 mRNA levels were also much higher than DGAT1 mRNA levels in DMSO-treated adipocytes. Third, DGAT2 mRNA was the major one in DMSO-treated mouse RAW264.7 macrophages. Fourth, the above results using TaqMan qPCR assay were confirmed by SYBR Green qPCR assay, although SYBR Green qPCR assay was not as sensitive as TaqMan qPCR assay as reported [32, 34]. Finally, DGAT family gene expression profiles in the cultured mouse cells were similar to those in the developing seeds of tung tree where DGAT2 mRNA levels in developing seeds were up to 20-fold and 9-fold higher than DGAT1 and DGAT3, respectively. The relative abundance of DGAT mRNAs in tung tree seeds is similar between this report and a previous report [25]. In addition, DGAT2 but not DGAT1 mRNA levels were increased by DMSO, consistent with the findings that DGAT2 gene expression is up-regulated by cottonseed extracts and gossypol in mouse macrophages [39]. Finally, our experimental results reported here are in agreement with data mining results using the UCSC Genome Browser, which showed that DGAT2 expression is higher than DGAT1 expression in human adipose tissues. DGAT1 and DGAT2 mRNA levels are 40.8 and 64.9 RPKM in subcutaneous adipose, whereas they are 33.9 and 76.5 RPKM in visceral adipose collected from 8555 tissue samples of 570 adult post-mortem individuals, respectively [54, 55].

It was unclear if the relative abundance of DGAT1 and DGAT2 mRNAs reflects their relative abundance at the protein levels. It has been difficult to detect DGAT proteins in mammalian and plant cells mainly due to lack of good antibodies and recombinant proteins [20, 56–58]. In addition, it could be hard to compare the relative expression levels of DGAT1 vs. DGAT2 proteins directly since there is no guarantee that both antibodies will have the same titer and react to the corresponding protein linearly. Finally, it is a long way to translate mRNA abundance to functionality because so many factors may affect the final activity of the protein, such as mRNA translation at the protein level, translocation of the protein to correct compartment of the cell, protein folding, turnover and post-translational modifications. Nevertheless, these areas are very important and certainly need to be investigated thoroughly in the future.

Furthermore, the relative expression levels of DGAT genes may be different among the cell types or cellular differentiation stages. For example, DGAT1 mRNA levels are increased during fasting and return to normal levels upon refeeding in white adipocyte tissue and liver but not in brown adipocyte tissue; whereas DGAT2 mRNA levels are decreased after fasting and increased after refeeding in white adipocyte tissue but unchanged in brown adipocyte tissue or liver [59]. It was also shown that 3 T3-L1 adipocytes display phenotypic characteristics of mainly white adipocytes but also brown and beige adipocytes [60]. In this study, we only evaluated DGAT mRNA levels in differentiated adipocytes after 4-h serum starvation. It would be interesting to investigate the expression patterns of DGAT mRNAs in cultured adipocytes after refeeding in the future.

TTP gene expression was much higher in macrophages than adipocytes, whereas DGAT gene expression was much higher in lipid-synthesizing adipocytes than macrophages. TTP protein regulates gene expression at the post-transcriptional level by binding to AU-rich elements of some mRNAs and destabilizing them. TNFα and GM-CSF mRNAs are stabilized in TTP deficient mice [61, 62]. Over-accumulation of these proinflammatory cytokines in TTP knockout mice results in a severe inflammatory syndrome including arthritis, autoimmunity, and myeloid hyperplasia [63, 64]. Up-regulation of TTP reduces inflammatory responses in macrophages [65].

## Conclusions

The results provide definitive evidence that DGAT2 gene expression is the major form at the mRNA level in both plant and animal cells tested in this study including tung tree seeds and mouse adipocytes and macrophages. Our conclusion was supported by results from three different cell types (mouse adipocytes, mouse macrophages, tung tree seeds), from different time-course ranging from 30 min to 24 h in animal cells and 1–11 weeks in developing seeds, and from both TaqMan and SYBR Green qPCR assays. This finding should be useful for targeted approach to regulating lipid/oil accumulation in plants and animals.

## Abbreviations

ADIPOQ: Adiponectin
DGAT: Diacylglycerol acyltransferases
DMEM: Dulbecco’s modified Eagle’s medium
DMSO: Dimethylsulfoxide
qPCR: Quantitative real-time PCR
RPL19b: 60S ribosomal protein 19b
RPL32: 60S ribosomal protein 32
TAG: Triacylglycerol
TTP: Tristetraprolin
ZFP36: Zinc finger protein 36
